# Treatment Guidelines for Hyponatremia: Stay the Course

**DOI:** 10.2215/CJN.0000000000000244

**Published:** 2023-06-28

**Authors:** Richard H. Sterns, Helbert Rondon-Berrios, Horacio J. Adrogué, Tomas Berl, Volker Burst, David M. Cohen, Mirjam Christ-Crain, Martin Cuesta, Guy Decaux, Michael Emmett, Aoife Garrahy, Fabrice Gankam-Kengne, John K. Hix, Ewout J. Hoorn, Kamel S. Kamel, Nicolaos E. Madias, Alessandro Peri, Julie Refardt, Mitchell H. Rosner, Mark Sherlock, Stephen M. Silver, Alain Soupart, Chris J. Thompson, Joseph G. Verbalis

**Affiliations:** 1 University of Rochester School of Medicine and Dentistry, Rochester, New York; 2 Rochester General Hospital, Rochester, New York; 3 University of Pittsburgh School of Medicine, Pittsburgh, Pennsylvania; 4 Baylor College of Medicine, Houston, Texas; 5 University of Colorado Aschutz School of Medicine, Aurora, Colorado; 6 University of Cologne Faculty of Medicine, Cologne, Germany; 7 Oregon Health and Science University, Portland, Oregon; 8 University of Basel, Basel, Switzerland; 9 Hospital Clinico San Carlos, Madrid, Spain; 10 Erasmus University Hospital, Brussels, Belgium; 11 Baylor University Medical Center, Dallas, Texas; 12 Tallaght University Hospital, Dublin, Ireland; 13 EpiCura Hospital, Ath, Belgium; 14 Erasmus Medical Center, Rotterdam, The Netherlands; 15 University of Toronto, Toronto, Ontario, Canada; 16 Tufts University School of Medicine, Boston, Massachusetts; 17 University of Florence School of Medicine, Florence, Italy; 18 University of Virginia School of Medicine, Charlottesville, Virginia; 19 RCSI School of Medicine, Dublin, Ireland; 20 Georgetown University Medical Center, Washington, DC

**Keywords:** complications, hypernatremia, hypokalemia, hyponatremia, malnutrition, mortality risk, osmolality, outcomes, risk factors, water–electrolyte balance

## Abstract

International guidelines designed to minimize the risk of complications that can occur when correcting severe hyponatremia have been widely accepted for a decade. On the basis of the results of a recent large retrospective study of patients hospitalized with hyponatremia, it has been suggested that hyponatremia guidelines have gone too far in limiting the rate of rise of the serum sodium concentration; the need for therapeutic caution and frequent monitoring of the serum sodium concentration has been questioned. These assertions are reminiscent of a controversy that began many years ago. After reviewing the history of that controversy, the evidence supporting the guidelines, and the validity of data challenging them, we conclude that current safeguards should not be abandoned. To do so would be akin to discarding your umbrella because you remained dry in a rainstorm. The authors of this review, who represent 20 medical centers in nine countries, have all contributed significantly to the literature on the subject. We urge clinicians to continue to treat severe hyponatremia cautiously and to wait for better evidence before adopting less stringent therapeutic limits.

## Introduction

For the past decade, most physicians treating severe hyponatremia have followed international guidelines that recommend limiting the rate of correction to avoid serious iatrogenic neurologic complications, most notably osmotic demyelination.^1,2^ Although these guidelines are based on small retrospective case series, they have been widely accepted by expert clinicians and are designed to minimize the risk of complications that can occur when correcting severe hyponatremia.^3–6^ Established practice has recently been challenged by the publication of a retrospective analysis of hospital administrative data derived from a cohort of over 17,000 patients hospitalized with hyponatremia.^7^ On the basis of the results of that study, an accompanying editorial suggested that hyponatremia guidelines have gone too far in limiting the rate of correction; its authors questioned the need for therapeutic caution and frequent monitoring of the serum sodium concentration.^8^ We believe such conclusions are unwarranted and potentially dangerous; they are reminiscent of a controversy that began many years ago. A review of that controversy, and of how current guidelines came to be, will explain the reason for our position.

## Historical Background

In the early 1980s, studies in experimental animals found that rapid correction of hyponatremia could induce brain lesions similar to those found in patients with central pontine myelinolysis (often referred to by its abbreviated name, CPM).^9,10^ These findings sparked a clinical controversy. Prominent experts at the time asserted that rapid correction of severe hyponatremia was needed for survival, that CPM was extremely rare and occurs in patients who were never hyponatremic, and that animal models and case reports in humans were flawed because brain lesions were found outside the pons and only after extremely large, rapid increases in serum sodium, often resulting in hypernatremia. Neurologic sequelae in patients with hyponatremia were attributed to hypoxia instead of a complication of therapy.^11,12^

In 1986, the term “osmotic demyelination syndrome” (often referred to by its abbreviated name, ODS) was introduced.^13^ Reporting on the course of eight patients who had developed clinical features of CPM after correction of severe hyponatremia (sodium ≤115 mmol/L and ≤105 mmol/L in four of the eight patients) by >12 mmol/L per day, the article suggested that neurologic complications of severe hyponatremia should be described in clinical rather than anatomical terms. The patients exhibited a stereotypical course characterized by gradual neurologic deterioration, with clinical features suggestive of CPM, beginning 3–6 days after partial or complete correction of severe, chronic hyponatremia. The clinical course was often biphasic: initial improvement in hyponatremic symptoms followed by a delayed onset of new neurologic findings. Because initial brain images were often negative, they were deemed confirmatory rather than essential for the diagnosis. A literature review of patients with sodium ≤105 mmol/L found that over half of those corrected by >12 mmol/L per day had developed neurologic complications after vigorous therapy—often after initially presenting with limited symptoms; half of those had CPM documented by imaging or autopsy, while in most of the others, CPM had been diagnosed clinically. By contrast, all 13 cases with sodium ≤105 mmol/L who had been corrected by <12 mmol/L per day recovered uneventfully.

In 1987, a study conducted in two large teaching hospitals using retrospective chart reviews of patients with sodium ≤110 mmol/L confirmed the conclusions of the literature review. Osmotic demyelination syndrome occurred in 13% of the 54 patients who had become hyponatremic outside the hospital while drinking conventional volumes of water (defined as chronic), while patients with self-induced water intoxication due to psychosis and patients who had become hyponatremic in the hospital (defined as acute) tolerated very rapid correction.^14^ In all patients with osmotic demyelination, the serum sodium had been increased by >12 mmol/L in 24 hours. In a subset with sodium ≤105 mmol/L, four of seven patients corrected by >12 mmol/L in 24 hours developed osmotic demyelination (57%), while all six corrected by <10 mmol/L in 24 hours recovered uneventfully.

Subsequently, several studies confirmed that osmotic demyelination was associated with more rapid rates of correction of chronic hyponatremia.^15–21^ Excluding case series on the basis of patient referrals, studies of patients with a sodium ≤120 mmol/L that include data on correction rates and outcomes are presented in Table 1. The frequency of osmotic demyelination varied widely among studies, depending on pretreatment serum sodium, correction rate, and method of identifying post-therapeutic neurologic sequelae. However, it is apparent that in patients with chronic hyponatremia, the lower the serum sodium and the larger the increase, the more likely that osmotic demyelination will be identified.

**Table 1:** Post-treatment neurologic sequelae in studies of patients with serum sodium concentrations ≤120 mmol/L

Author	Year	Number of Patients	SNa (mmol/L)/Study Method	Post-Treatment Sequelae Overall	Post-Treatment Sequelae with Rapid Correction	Documented CPM or EPM by Imaging or Autopsy	Definition of Rapid Correction (mean values in patients with sequelae in studies without defined criteria)
Sterns^14^	1987	64	≤110 Retrospective	7 (11%)	7/42 (17%)	1	>12 mmol/L/24 h
Brunner *et al*.^15^	1990	13	<115 (93–113) Prospective MRIs	3 (23%)	3/13 (23%)	3	30±9.6 mmol/24 h
Tanneau *et al*.^21^	1994	84	≤115 Retrospective	5 (6%)	?	1	21.8±3.9 mmol/L/24 h
Ellis^16^	1995	184	≤120 Prospective evaluation by neurologist	9 (5%)	8/56 (14%)^a^	2	>10 mmol/L/24 h
Vu *et al*.^17^	1995	255	≤120 Retrospective	4 (1.5%)	4/37 (10.8%)	4	>12 mmol/L/24 h
Nzerue *et al*.^20^	2003	168^b^	<115 Retrospective	1 (0.6%)	1/17 (6%)^b^	0	>25 mmol/L/48 h
Geogheghan *et al*.^18^	2015	412	<120 Retrospective	1 (0.24%)	1/114 (1%)	1	>8 mmol/L/24 h
George *et al*.^19^	2018	1490	<120 Retrospective	8 (0.5%)	7/606 (1.2%) 6/390 (1.5%)	8^c^	606 corrected by >8 mmol/L/24 h 390 corrected by >12 mmol/L/24 h

SNa, serum sodium; CPM, central pontine myelinolysis; EPM, extrapontine myelinolysis.

a Daily serum sodium values were not measured in one patient with sequelae.

b Eighty-two percent of patients had acute hyponatremia (<48 h); percentage of rapidly corrected patients with chronic hyponatremia not reported.

c One patient had been treated at another hospital for serum sodium of 105 mmol/L before admission.

When osmotic demyelination syndrome was first described, it was widely believed that to avoid potentially fatal neurologic complications of severe symptomatic hyponatremia, it was important to rapidly raise the serum sodium to a “safe” level above 120 mmol/L and, according to some experts, to as high as 130 mmol/L.^11,12,22^ At a sodium ≤105 mmol/L, it becomes impossible to both raise the serum sodium to a “safe” level and avoid correction by >12 mmol/L per day; a choice must be made between the risks of persistent, severe hyponatremia, and the risks of osmotic demyelination.^23^ Because a sodium ≤105 mmol/L is uncommon, a letter was sent to all members of the American Society of Nephrology seeking data on patients with a sodium ≤105 mmol/L who had been recently treated, regardless of outcome.^24^ Data on 56 patients obtained from 40 medical centers confirmed that outcomes depend on the chronicity of hyponatremia, as previously defined^14^: While none of the 18 patients with acute hyponatremia suffered complications, regardless of correction rate, 14 of 28 patients with chronic hyponatremia corrected by >12 mmol/L in 24 hours developed post-therapeutic neurologic complications; in most, the clinical course was consistent with CPM (a delayed onset of neurologic symptoms beginning 2–6 days after initial improvement), but only three had CPM documented by imaging. The amount of correction over 48 hours was also associated with post-therapeutic complications: 14 of 27 chronic patients (52%) corrected by >18 mmol/L in 48 hours were affected. All patients corrected by >12 mmol/L in 24 hours had also been corrected by >18 mmol/L in 48 hours, and all but one corrected by <12 mmol/L in 24 hours were corrected by <18 mmol/L in 48 hours.

Many published series and case reports have reported similar findings in patients treated for severe, chronic hyponatremia.^4,25^ A variety of neurologic findings can develop after a large, rapid increase in serum sodium: seizures; swallowing dysfunction, often with aspiration; dysarthria; motor weakness or paralysis; tremors and movement disorders; oculomotor dysfunction; and behavioral disturbances. These deficits reflect demyelination in the central pons (central pontine myelinolysis) and/or similar symmetrical lesions outside the pons (extrapontine myelinolysis or EPM).^26^ In many cases, lesions can be found on MRI, but they are usually missed by computed tomography scans (CT). Typically, the MRI is normal at the onset of post-therapeutic neurologic findings, and lesions may not become evident until weeks later. In some cases, post-therapeutic neurologic findings resolve over a few days or weeks, and no lesions are ever documented by MRI.^4^ Autopsy-defined or MRI-defined cases represent the most severe end of the spectrum of injury caused by excessive correction. More subtle injury is more difficult to identify and may occur at less rapid correction rates.^27^

Osmotic demyelination syndrome constitutes a subset of CPM or EPM. These brain lesions can occur for reasons other than rapid correction of hyponatremia, and the syndrome can occur without demonstrable lesions of myelinolysis. Other osmotic insults, such as hypernatremia and severe hyperglycemia, have been reported to result in myelinolysis, without the typical biphasic clinical course of osmotic demyelination syndrome^28^; myelinolysis can be induced by hypernatremia in experimental animals.^29^ Because myelinolysis can also develop in the absence of osmotic stress (hyperammonemia, thiamine deficiency, and malignancies), it may not be appropriate to ascribe positive brain images to minor changes in serum sodium if clinical features of osmotic demyelination syndrome are absent, particularly in patients with sodium >115 mmol/L.^30^

Studies of experimental hyponatremia in rats, dogs, rabbits, and mice have confirmed that correction of hyponatremia rather than hyponatremia itself is the cause of brain demyelination.^31,32^ Ultrastructural damage to glial cells can be documented within hours after the increase in serum sodium, triggering delayed cell death followed by breakdown of the blood–brain barrier and demyelination.^33^ Relowering of the serum sodium immediately after a rapid increase can abort the process, preventing demyelination.^34^ Case reports in humans suggest that therapeutic relowering of the serum sodium after rapid correction of hyponatremia is well tolerated and possibly beneficial.^35,36^

## Practice Guidelines

After weighing the evidence, the European Clinical Practice Guidelines recommended that correction of hyponatremia be limited to 10 mmol/L in the first day of treatment and 8 mmol/L for every subsequent day thereafter.^1^ An expert panel that included six physicians from the United States and one from Ireland (which we will call the “US/Irish expert panel”) came to similar conclusions, but with some added nuance (expressed in italics).^2^ For *chronically* hyponatremic patients *with a sodium ≤120 mmol/L* (for example, outpatients drinking conventional volumes of water or treated with thiazides and hospital-acquired hyponatremia with a known duration >48 hours) who were at *normal risk* of developing osmotic demyelination, the panel recommended a correction limit of 10–12 mmol/L in *any* 24-hour period and 18 mmol/L in *any* 48-hour period and a *minimum* correction of 4–8 mmol/L. The panel recommended *increased vigilance* for patients with a sodium ≤120 mmol/L at *heightened risk* of osmotic demyelination. Factors reported to be associated with a higher risk of osmotic demyelination include sodium ≤105 mmol/L, alcohol use disorder, hypokalemia, malnutrition, or advanced liver disease. In these *high-risk patients*, the US/Irish expert panel recommended that correction should not exceed 8 mmol/L in *any* 24-hour period and that the minimum daily correction goal should be 4–6 mmol/L. In patients without major risk factors for osmotic demyelination, the panel noted that correction by 8–12 mmol/L in the first day of therapy was greater than necessary, but unlikely to cause harm as long as the 2-day increment does not exceed 18 mmol/L.

For patients presenting with severe symptoms, the European Clinical Practice Guidelines and US/Irish expert panel both advocate bolus infusions of hypertonic saline in an effort to raise the serum sodium by 5 mmol/L (European) or by 4–6 mmol/L (US/Irish) within a few hours. An increase of this magnitude is sufficient to markedly reduce intracranial pressure and can reverse impending brain herniation.^37^ Bolus infusion of hypertonic saline has been reported to achieve the desired increment in serum sodium more rapidly than a continuous infusion of 3% saline,^38^ and it is associated with better clinical outcomes.^39^

After treatment with either isotonic saline or hypertonic saline given by bolus or continuous infusion, the serum sodium often increases by more than intended.^19,27,39,40^ Indeed, the risk of “overshooting the mark” is one of the stated reasons for setting conservative correction goals when treating patients for severe hyponatremia.^41^ However, the main reason for an excessive rise in serum sodium is not an excessive dose of saline; rather, it is the sudden elimination of a large volume of dilute urine.^2,13,14,42^ If the cause of water retention and hyponatremia resolves, a water diuresis (often called an aquaresis) will emerge during treatment and can increase serum sodium by more than 2 mmol/L per hour.^2,42,43^ For this reason, in patients with a sodium ≤120 mmol/L, it is important to measure the serum sodium frequently and to monitor urine output carefully during treatment.

Analytical limitations of serum sodium measurements must also be considered when setting correction goals and limits.^44^ Because of unavoidable imprecision, a laboratory report indicating that the serum sodium has increased by 8 mmol/L might, in reality, reflect an increase of 10 mmol/L. For that reason, when planning therapy, the targeted rate of correction should not be too close to rates that can result in patient harm.

Administration of desmopressin (a synthetic antidiuretic hormone) either after or in anticipation of a water diuresis has been used to reverse or prevent inadvertent excessive correction of hyponatremia.^42,45,46^ Both the European Clinical Practice Guidelines and the US/Irish expert panel suggest that relowering of the serum sodium should be considered if correction limits have been exceeded.^1,2^ However, the approach should depend on the relative risks of injury from excessive correction. The strongest case for relowering the serum sodium to prevent osmotic demyelination can be made in patients at very high risk of developing the disorder (those with a sodium ≤105 mmol/L or patients with heavy alcohol use, severe hypokalemia, malnutrition, or advanced liver disease). It would be reasonable to relower the serum sodium in such patients if it has increased by >8 mmol/L in <24 hours. On the other hand, patients who have rapidly become hyponatremic due to self-induced water intoxication related to psychosis or endurance exercise often develop a spontaneous water diuresis that rapidly brings the serum sodium back to normal; because their risk of osmotic demyelination syndrome is extremely low, the US/Irish expert panel deemed efforts to prevent or reverse excessive correction to be unnecessary. Similarly, in most patients presenting with a sodium >120 mmol/L, relowering the serum sodium after correction by >8 mmol/L or even >12 mmol/L was not recommended. In patients with a sodium ≤120 mmol/L without major risk factors for osmotic demyelination, initial correction by 8–12 mmol/L is more than necessary, but unlikely to be harmful unless the 48-hours limit of 18 mmol/L is exceeded; the panel deemed relowering of the serum sodium to be optional after correction by >10–12 mmol/L but recommended that further increases in serum sodium should be avoided for the next 24 hours. Other experts have adopted the stricter limit of 8 mmol/L in 24 hours for all patients.^3^

Inadvertent overcorrection can be avoided by replacing urinary water losses or by stopping them with desmopressin (DDAVP). Alternatively, desmopressin can be given at the beginning of therapy and at regular intervals thereafter to keep the urine concentrated until the serum sodium has been gradually returned to near normal levels by concurrent administration of hypertonic saline given as a slow continuous infusion or small titrated boluses.^46,47^ The technique, which has been called the “DDAVP clamp,” has been reported to successfully meet correction goals, but more data are needed comparing the technique to other therapeutic options.

Some experts have never accepted the term “osmotic demyelination syndrome,” asserting that limits recommended by current guidelines are too strict, potentially deterring physicians from providing life-saving therapy with hypertonic saline.^48^ They have maintained since the beginning of the controversy that neurologic complications from untreated or undertreated hyponatremia (seizures, respiratory arrests, and herniation) are more common than neurologic complications due to excessive therapy.

## Recent Revival of the Controversy

Support for the idea that osmotic demyelination syndrome is extremely rare was provided by uncritical acceptance of the results of a recent, large retrospective study; the investigators concluded that myelinolysis affects only 0.05% of patients with hyponatremia and is unrelated to rapid correction (defined as an increase of >8 mmol/L per day).^7^ However, nearly 90% of the patients studied had a sodium >120 mmol/L, and others may have had acute hyponatremia due to self-induced water intoxication or exacerbation of hyponatremia by hyperglycemia. Although the risk of osmotic demyelination syndrome in such patients is known to be vanishingly low, the study weighed the benefits of a therapeutic limit that was intended for patients whose risk of osmotic demyelination syndrome was unusually high.^2^

Adherence to an 8-mmol/L daily correction limit in patients with a serum sodium >120 mmol/L would reflect an abundance of caution^41^; but, predictably, if that limit was marginally exceeded, very few patients would be harmed. Notwithstanding, myelinolysis was documented in 2.6% of patients in a subset of the study's population with a sodium <110 mmol/L, a population that has been shown to be at higher risk for osmotic demyelination syndrome when rapidly corrected.^14^ But even that figure likely underestimates the true prevalence of osmotic demyelination syndrome among such patients because of the method used to identify patients with the disorder. To identify patients with osmotic demyelination syndrome, this study relied on diagnostic coding of medical records and neuroimaging reports during the index hyponatremia admission and during readmissions within 7 days. However, as discussed above, osmotic demyelination syndrome is a clinical, not a radiologic, diagnosis, with varying severity. Symptoms of osmotic demyelination syndrome are delayed, often leading to readmission more than a week after discharge, if at all. Lesions consistent with osmotic demyelination syndrome are seldom evident on MRI at the onset of symptoms and may not become positive for weeks, if at all.

The study's conclusion that osmotic demyelination syndrome is unrelated to correction of hyponatremia was based on the finding that most patients with myelinolysis had been corrected by <8 mmol/L per day. However, as many as five patients with an initial sodium of 126–129 mmol/L developed demyelinating brain lesions despite an initial correction <8 mmol/L in 24 hours. Subsequently, all developed hypernatremia to the levels of 153–164 mmol/L, indicating a 24–38 mmol/L increase in serum sodium. These findings actually support a relationship between osmotic insults and demyelinating brain lesions. When a diagnosis of CPM or EPM is made and there is no documentation that correction rates ever exceeded 8 mmol/L per day, there is a possibility that correction of hyponatremia had already begun before the patient's admission to the hospital.

An accompanying editorial, commenting favorably on the study,^8^ cited two other recent studies with “similar findings” to support its conclusions (Table 1).^18,19^ However, the incidence of osmotic demyelination syndrome in the two studies was more than ten-fold higher, and their authors came to conclusions that differed considerably from those expressed by the editorial. The authors of one study acknowledged that while only 0.5% of the 1490 patients with a sodium <120 mmol/L corrected by >8 mmol/L per day (and 1.5% of 390 corrected by >12 mmol/L per day) had developed osmotic demyelination syndrome (Table 1), it was possible that some cases with osmotic demyelination syndrome had been missed.^19^ The authors of the other study speculated that a possible reason for the low incidence of osmotic demyelination syndrome at their medical center (1% of 114 patients with sodium <120 mmol/L corrected by >8 mmol/L per day) was that limits were only marginally exceeded.^18^ As mortality rates were not higher when the first day's correction was ≤5 mmol/L, they discouraged clinicians from exceeding established limits. Other investigators have reported a dramatic fall in mortality from severe hyponatremia in recent years, despite a cautious approach to avoid excessive correction.^49^

Our treatment of hyponatremia should be informed by the best possible data, addressing two critical questions: How much correction is enough and how much is too much? Valid answers to these questions require studies of populations at high risk of complications from both excessive and inadequate correction of hyponatremia—patients with very low serum sodium. A study of individuals diagnosed with MRI-documented myelinolysis in the Swedish National Patient Register during 1997–2011 identified 83 patients with the disorder; 86.7% of patients were hyponatremic (all chronic), with a median sodium level at admission of 104 mmol/L, and all but six had been corrected by >8 mmol/L in 24 hours.^50^ To be reliable, such studies will require rigorous methods to quantify increases in serum sodium throughout the hospital course and to accurately identify all complications—not just the most severe. In the future, those methods might include sophisticated software. But for now, meticulous chart review by hand, the method used when “osmotic demyelination syndrome” was given its name 37 years ago, remains the best approach.

Thankfully, changes in practice patterns have made osmotic demyelination syndrome less common than it was in the 1980s. On the basis of what we know today, correction of a sodium ≤120 mmol/L by >10 mmol/L within 24 hours or by >18 mmol/L within 48 hours should be avoided—not because raising the serum sodium too rapidly *commonly* causes osmotic demyelination syndrome but because it *can* cause the syndrome. It has always been clear that overly rapid correction of hyponatremia does not inevitably lead to osmotic demyelination; rather, it is associated with a higher risk of osmotic demyelination.

The longer the duration of hyponatremia and the lower the serum sodium, the greater the risk for injury from excessive correction. If the sodium is ≤105 mmol/L or if there are additional risk factors for osmotic demyelination syndrome (alcohol use disorder, hypokalemia, malnutrition, or advanced liver disease), correction by >8 mmol/L per day should be considered excessive. The cost of limiting correction (more frequent blood draws and perhaps a somewhat longer hospitalization) is trivial when compared with the devastating consequences that can affect even surviving patients with osmotic demyelination syndrome. We strongly believe that abandoning established safeguards now is a bit like discarding your umbrella because you have remained dry in a rainstorm. We urge clinicians to continue to treat severe hyponatremia cautiously and to wait for better evidence before adopting less stringent therapeutic limits.
